# From disagreements to dialogue: unpacking the Golden Rice debate

**DOI:** 10.1007/s11625-018-0577-y

**Published:** 2018-05-17

**Authors:** Annika J. Kettenburg, Jan Hanspach, David J. Abson, Joern Fischer

**Affiliations:** 1 Faculty of Sustainability, Leuphana University of Lüneburg, Universitätsallee 1, 21335 Lüneburg, Germany; 2 Lund University Centre for Sustainability Studies (LUCSUS), Lund University, Box 170, 22100 Lund, Sweden

**Keywords:** Cluster analysis, Disciplinary divide, Food security, Genetically modified crops, Problem framing, Sustainability science

## Abstract

Transgenic Golden Rice has been hailed as a practical solution to vitamin A deficiency, but has also been heavily criticized. To facilitate a balanced view on this polarized debate, we investigated existing arguments for and against Golden Rice from a sustainability science perspective. In a structured literature review of peer-reviewed publications on Golden Rice, we assessed to what extent 64 articles addressed 70 questions covering different aspects of sustainability. Using cluster analysis, we grouped the literature into two major branches, containing two clusters each. These clusters differed in the range and nature of the sustainability aspects addressed, disciplinary affiliation and overall evaluation of Golden Rice. The ‘biotechnological’ branch (clusters: ‘technical effectiveness’ and ‘advocacy’) was dominated by the natural sciences, focused on biophysical plant-consumer interactions, and evaluated Golden Rice positively. In contrast, the ‘socio-systemic’ branch (clusters: ‘economic efficiency’ and ‘equity and holism’) was primarily comprised of social sciences, addressed a wider variety of sustainability aspects including participation, equity, ethics and biodiversity, and more often pointed to the shortcomings of Golden Rice. There were little to no integration efforts between the two branches, and highly polarized positions arose in the clusters on ‘advocacy’ and ‘equity and holism’. To explore this divide, we investigated the influences of disciplinary affiliations and personal values on the respective problem framings. We conclude that to move beyond a polarized debate, it may be fruitful to ground the Golden Rice discourse in facets and methods of sustainability science, with an emphasis on participation and integration of diverging interests.

**Electronic supplementary material:**

The online version of this article (10.1007/s11625-018-0577-y) contains supplementary material, which is available to authorized users.

## Introduction

Sustainability is a contested and highly normative concept (Dobson 1999; Christen and Schmidt 2012). The solution-oriented field of sustainability science (Miller et al. 2014) has to address both the normative goals of sustainability itself and the, often implicit, assumptions that underpin different scientific traditions (Schumpeter 1954; Funtowicz and Ravetz 1993; Lélé and Norgaard 2005). Such normativity, especially when not explicitly addressed, often leads to conflicting, even polarized, discourses regarding what represents an appropriate intervention for a given sustainability problem. For example, polarized narratives in research addressing the intersecting goals of food security and biodiversity conservation are driven by the underpinning conceptualization of the problem as either technical or socio-political (Loos et al. 2014; Glamann et al. 2015). Similarly, the narrative explaining food insecurity as a result of insufficient production and population growth contrasts with explanations based on unequal distribution of social power as well as economic and physical resources (Sen 1981; Legwegoh and Fraser 2015). In the agricultural biosciences, calls for gene patenting, corporate funding of public institutions and public–private partnerships conflict with arguments that seeds should be regarded as public goods (Scoones 2002; Stone 2015). Such polarization presents serious challenges for sustainability science, not simply in terms of conflicting policy prescriptions, but also in the perceived legitimacy of the science itself (Bäckstrand 2003).

In this paper we use the example of the scientific discourse around “Golden Rice”—itself a microcosm of the broader debate surrounding the role of genetically modified organisms (GMOs) in agricultural sustainability—as a particularly emotive example of a polarized discourse in sustainability science. Through a systematic, quantitative (cluster) analysis of the scientific literature, we classify and describe the polarized positions within the Golden Rice debate. By viewing this discourse through an explicit sustainability lens we seek to shed light on the role of problem framing in shaping the Golden Rice discourse, and suggest ways of shifting from such polarized debates towards more constructive dialogues. In particular, we highlight the importance of understanding and acknowledging the sources of such polarization, to move beyond ‘siloed’ disagreements towards shared understandings and meaningful solutions.

The severity of conflicts around the use of GMOs in agriculture has been likened to that of a war (Lang and Heasman 2004; Waltz 2009; Stone 2015). Proponents see in genetically modified (GM) crops powerful tools to increase yields (Carpenter 2010), improve crop quality, decrease pesticide use (Christou et al. 2006), fight micronutrient deficiencies, adapt plants to climate change and facilitate economic growth (Phillips 2002). Opponents voice doubts over the long-term effectiveness of genetically modified crops in face of accelerated formation of resistances to glyphosate (Gilbert 2013) and to Bt toxins (Tabashnik et al. 2013), over the nutritional equality to non-GM crops (Bøhn et al. 2014), and even over their adequateness as food and feed (Séralini et al. 2014). Some call into question genetic engineering’s theoretical foundation on reductionist models that disregards insights from systems biology (e.g., McAfee 2003; Perret and Longo 2016). Often these GMO specific issues are entangled with political concerns regarding the role of GMOs in reinforcing corporate power (Walters 2005), or the promotion of monocultures and homogenization of diets and landscapes (Scrinis 2007).

The case of Golden Rice exemplifies many of the conflicts surrounding GMOs as a potentially sustainable solution for issues ranging from food security to biodiversity conservation. Golden Rice is a genetically modified cultivar that synthesizes beta-carotene, which in turn is metabolized into vitamin A in the human body. Some communities in the Global South show high rates of xerophthalmia, the clinical manifestation of vitamin A deficiency (Thylefors et al. 1995). Xerophthalmia leads to corneal ulceration and ultimately blindness. An estimated 250,000–500,000 vitamin A-deficient children became blind every year in the period 1995–2005, half of them dying within 12 months of losing their sight (WHO 2017). However, data are largely outdated; only 27 countries reported estimates since 2006 (Wirth et al. 2017). Vulnerability to xerophthalmia depends on a number of factors including eating habits such as a varied diet accompanied by fats, social determinants such as poverty or lack of education, health conditions such as parasitic infestations, and access, influenced by seasonality of vitamin A rich vegetables/fruits, land entitlements, and crisis such as famine or flight (Egana 2003; Oyunga et al. 2016). Current strategies to address vitamin A deficiency include supplementation, (bio-)fortification and dietary diversification (Ruel 2001; WHO 2017).

Golden Rice was developed by Potrykus and Beyer in Zürich and Freiburg during the 1990s in response to a call from the Rockefeller Foundation for a plant breeding solution to vitamin A deficiency. This work resulted in the first novel rice variety, which contained 1.6 µg/g carotenoids in the endosperm. The second generation of Golden Rice, created in partnership with Syngenta, contains up to 37 µg/g carotenoids, sufficient to fulfil half of daily vitamin A requirements with 60 g of uncooked rice (Paine et al. 2005).^1^ Syngenta agreed on free licenses for famers in the Global South with incomes less than $10,000 annually. These farmers may reseed Golden Rice after every harvest. Currently, Golden Rice is still under development, with the intention that once all safety assessments are completed and it is approved by national regulators, it will be distributed accompanied by information campaigns (Potrykus 2001; Mayer and Potrykus 2011; Zeigler 2014).

Golden Rice promises a positive impact on human health while ensuring economic independence of smallholder farmers from large agri-business. Nevertheless divergent views regarding the benefits and sustainability of Golden Rice persist (e.g., Small 2014). Here, we present a systematic, quantitative assessment of the narratives in the peer-reviewed discourse on Golden Rice. Our objectives were to (1) identify and characterize narrative-based groups of articles on Golden Rice, (2) point out the scope of themes relevant to sustainability addressed by each group, and (3) propose explanations for the revealed patterns. Drawing on this, we provide ideas to facilitate a more fruitful dialogue within and beyond the scientific community regarding Golden Rice. Although our case study is specifically on Golden Rice, our approach to understanding and resolving this contentious scientific debate may also help to inform the analysis of other polarized discourses on pathways to sustainability.

## Methods

### Literature selection

We conducted a literature review in Scopus of English language, peer-reviewed articles and book chapters using the keyword “Golden Rice” in title–abs–key in July 2016. An article was included in the analysis if it met the following criteria. Criterion one: Golden Rice was discussed as both a biotechnological project and a health intervention, either as the focus of the paper or within a broader framing. Just mentioning Golden Rice as an illustrative example led to exclusion [e.g., in Weil (2005) “Are genetically modified plants useful and safe?”]. Criterion two: articles focusing on biophysical and technical matters only were excluded (e.g., Al-Babili et al. 2006). Criterion three: the article addressed three or more questions of our coding protocol. This minimum level was set to guarantee the validity of quantitative results. In contrast to the first two criteria, criterion three was applied after coding of the article.

### Identification of sustainability themes

Our intention was to offer a sustainability perspective on the Golden Rice debate: which sustainability themes do the different strands of Golden Rice literature address? We defined sustainability as an ideal of human well-being within planetary boundaries across generations (Gibson 2006; Rockström et al. 2009). To operationalize this definition, we identified 16 themes, which, arguably, ought to be considered in discussions about Golden Rice from a sustainability perspective (Table 1). In a second step, we used an inductive approach to identify specific questions (sub-themes) under each sustainability theme that emerged from the reviewed articles. A grounded theory-based, inductive formulation and adjustment of questions during the coding process (Corbin and Strauss 1990) provided a higher thematic coverage of sustainability sub-themes, adding new questions and dismissing unaddressed ones. The final coding protocol resulted in 70 questions/sub-themes (Table S1 in the supplementary material). Those 70 sub-themes were coded for text analysis in MAXQDA 12 (VERBI Software 2016).

**Table 1:** Description of the sustainability ‘themes’ and their relevance for the Golden Rice discourse

Theme	Foundation from sustainability science
Participation	Participation of people/groups/institutions affected by and affecting vitamin A deficiency increases the legitimacy of the research process and provides opportunities for mutual learning. Exchange and cooperation among actors are keys to societal changes that confront deep causes of unsustainability (Fischer et al. 2012; Lang et al. 2012; Reed et al. 2016). Is the picture of vitamin A deficiency and its causes drawn with help of local voices, were they included in the intervention design or knowledge creation process (Chambers 1995)?
Local culture	Influences on the acceptance of Golden Rice, such as the cultural value of white rice, traditions of how to feed children and openness to innovations, shape the likelihood of its success (Jolivet and Maurice 2006; Thurber and Fahey 2009). Furthermore, the intercultural gap between scientists and local communities has caused failure of projects in the past (Trickett 2011; Minasyan 2015)
Health and well-being	Do authors have a clear understanding of the predictors of vitamin A deficiency, enabling them to position technical strategies within a broader range of possible interventions (Ruel 2001; Oyunga et al. 2016)? Do they acknowledge the frequent co-occurrence of multiple deprivations and their reinforcing interrelations (Black et al. 2003; Olsson et al. 2014)?
Dignity and human rights	Are people suffering from Vitamin A deficiency recognized as autonomous individuals with rights to and control over their food system, as advocated by the food sovereignty movement (Wittman 2009; Perfecto et al. 2009)?
Equity and empowerment	Health and life quality are, among others, related to socio-economic status, gender and race; therefore, a comprehensive health strategy must also aim at reducing inequality and seeking social justice (Olsson et al. 2014). This includes empowerment through education and capacity building, especially of women and children. In addition, making an informed choice on Golden Rice requires not only balanced information, but also the capacity to evaluate it critically (Valente et al. 1998; Kent 2005)
Actors, resources and power	To determine who will profit to what extent from Golden Rice, actor constellations and their resources must be understood, followed by an investigation of how Golden Rice might act within and change these relations and resource pools (Babcock and Francis 2000; Cloke 2013)
Governance and institutions	Formal and informal institutions determine people’s nutrition via cultural habits, supply chains and global trade mechanisms such as liberalization, protectionism, and food speculation (Cannon 2002). Do articles address good governance, which presents an opportunity for mediating power disparities (Sayer et al. 2014)?
Climate change	Irrigated rice agriculture accounts for a variable but significant amount of methane emissions (Mosier et al. 2001). At the same time, Golden Rice fields and farming will be subject to considerable climate variations, which pose the risk of food shortages (Patz et al. 2005; Dixon et al. 2009)
Water and soil conservation	What impact will Golden Rice have on abiotic components of the ecosystem? Will it need fertilizer, pesticides and irrigation? Designing a new rice variety offers a chance to address the disturbance of nitrogen and phosphorus cycles and scarcity of drinking water (IAASTD 2009)
Biodiversity	Like prior green revolution rice varieties, Golden Rice is likely to be cultivated in monocultures. This implies adverse effects on local ecosystems like a loss of biodiversity (Stone and Glover 2016). Biodiversity has a potential to function as win-win-situation, improving both ecosystem and human health through sustainable farming methods such as agroecology (Sayer et al. 2013; Fischer et al. 2017)
Resilience and risks	Acknowledging unclear dynamics of systems, both in short and long terms, acts against reductionism and promotes diversified strategies to buffer partial failure (Walker et al. 2004; Janssen et al. 2007). How will Golden Rice act on a biophysical level, within a social system or when confronted with disturbance? Are uncertainties made transparent?
Holism and systems thinking	The rollout of Golden Rice might enforce certain developments through positive feedback or create lock-ins, e.g., by influencing the GM crops market and regulation schemes (Ericksen 2008; Vanloqueren and Baret 2009; Cairns 2014). Referring to system archetypes, Golden Rice might be characterized by a ‘shifting the burden’ model, a quick fix yielding good results and reducing the perceived urgency to change underlying poverty and environmental degradation in the long-term (Banson et al. 2016). Apart from serving as an ontological concept, systems thinking functions as epistemological lens to perceive interconnectedness, synergies and cross-scale-dynamics (Abson et al. 2016)
Cost-utility-analysis	Are costs and efficiency of Golden Rice compared to other measures against VAD? A comprehensive evaluation of Golden Rice and its alternatives would need to consider opportunity costs, external effects and whole life cycles in monetary and non-monetary terms (Cohen and Winn 2007)
Achievability and realization prospects	Are information and distribution strategies thought of in detail? One characteristic of rural low income people is the difficult accessibility of their homes, often accompanied by minimal access to media. Further to consider are e.g., the provision of human and financial resources, institutional support, possible value discrepancies and the design of monitoring programs (Sayer et al. 2014)
Values and transparency	Do authors make normative judgements, e.g., on the precautionary principle? Are values made transparent when formulating recommendations and evaluating risks, as well as when framing the problem and designing the research agenda? Transparency and self-disclosure are keys to provide good science (Staddon 2001; Devos et al. 2014)
Philosophy and reflection	Is the role of science and technology in solving complex societal problems addressed? Such consideration helps to determine the chances and limitations of Golden Rice and to understand the philosophical base Golden Rice is embedded in – an opportunity for deeper reflection about our society and the causes for unsustainability (Vanloqueren and Baret 2009; Scott 2011; Garnett 2013)

These themes formed the basis for the selection of the 70 sub-themes used in the coding protocol (Table S1 in supplementary material)

We summarized the coding results quantitatively in a table. A paper scored 0 if it did not address a question at all and 1 if it addressed it. The intention of this quantitative coding was to differentiate alternative discourses related to the 16 key sustainability themes (Table 1) and the related 70 sub-themes (supplementary materials).

### Data analysis

After coding we used agglomerative hierarchical cluster analysis, a method commonly employed to recognize subsets in multivariate data. Agglomerative clustering begins with discontinuous single objects (i.e., articles) and groups these into ever larger clusters. Euclidean distance was chosen as an association measure for the clusters due to our homogeneous scale, the limited number of double zeros in pairwise comparisons (i.e., no answers) and the clear interpretation of the resulting patterns. We employed Ward’s minimum variance method for grouping. It minimizes the within-cluster sum of squared errors, that is the sum of the squared distances among cluster members divided by the number of articles per cluster, thereby usually producing clear and evenly sized clusters (Borcard et al. 2011). Importantly, cluster analysis is an exploratory method that is able to uncover (dis-)similarities between papers and thus to empirically show different schools of thought in the assessed literature. Therefore, unlike in the use of inferential statistics the potential lack of independence of data points (e.g., papers written by the same authors are not independent) does not invalidate or bias our analysis. Results of the cluster analysis were visualized in a dendrogram. Furthermore, we conducted an indicator analysis that listed the questions of central importance for each cluster, facilitating cluster characterization and differentiation (Dufrene and Legendre 1997). Analyses were performed in R Version 3.3.2 (R Core Team 2016).

## Results

The literature search returned 98 articles (after removing duplicates, non-peer-reviewed and inaccessible publications), of those 64 passed our inclusion criteria. Of the 34 excluded articles, 56% focused on a different topic (criterion one), 29% were entirely biochemical or technical (criterion two) and 15% addressed two or less questions (criterion three). Of the 64 reviewed articles most were authored by plant scientists including biologists and chemists (44%), followed by social scientists (25%) and economists^2^ (19%; Table 2). Forty-two percent of the articles were authored by members of the Golden Rice Humanitarian Board and affiliated research institutes, or by employees of Syngenta or Monsanto. Seventy-seven percent of all articles were in favour of Golden Rice whereas 14% voiced doubts or opposed it, and 9% abstained from judgement. Evaluation of Golden Rice (Table 2) ranged from outright rejection (opposing), through pointing to serious concerns (doubtful), arguing that there is insufficient data to draw conclusions (cautious), ascribing great potential to Golden Rice if confounding factors can be overcome (optimistic), to arguing for the immediate use of Golden Rice to avoid preventable deaths (passionate). The first paper was published in 2001 and numbers of publications per year were relatively steady (mean 4, SD 2.2).

**Table 2:** Information on message, discipline, affiliation and origin of selected articles (*n* = 64)

	Total	%
Message		
Optimistic	32	50
Passionate	17	27
Doubtful/cautious	8	12
Opposing	5	8
Neutral	2	3
Discipline		
Plant science	28	44
Social science	16	25
Economics	12	19
Interdisciplinary	6	9
Others	2	3
Affiliation		
Not part of GR board	39	61
Members of GR board	21	33
Corporate	4	6
Origin		
Global North	54	85
Mixed team	6	9
Global South	4	6

**Fig. 1:**
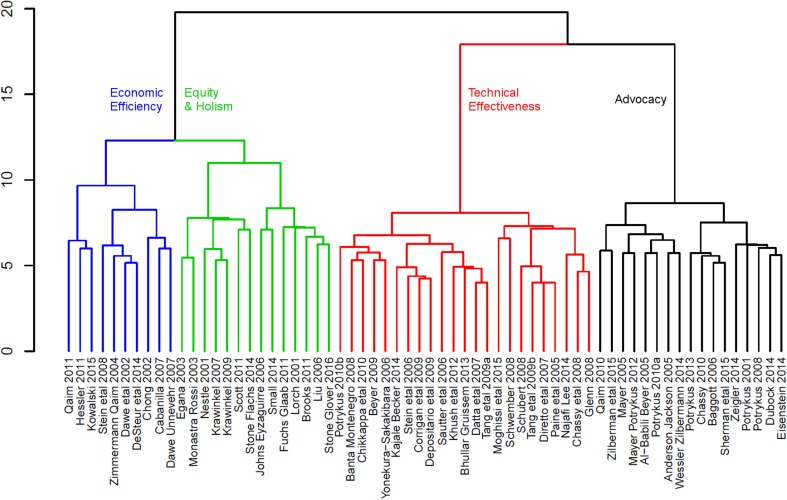
Cluster analysis demonstrated two major branches of research on Golden Rice, each consisting of two clusters—a biotechnical branch (red ‘technical effectiveness’, black ‘advocacy’) versus a socio-systemic branch (blue ‘economic efficiency’ and green ‘equity and holism’). For full citations see Table S3 in the supplementary material

Cluster analysis led to two major branches containing two clusters each (Fig. 1). The clusters differed significantly among each other not only in the range of sustainability sub-themes addressed (Table 3), but also in their subjective evaluations of Golden Rice. Moreover, there was a clear disciplinary divide between the clusters. The clusters were named to reflect their thematic focus: the biotechnological branch consisted of clusters on ‘technical effectiveness’ and ‘advocacy’, whereas the socio-systemic branch included clusters on ‘economic efficiency’ and ‘equity and holism’. In the following, we describe the branches and clusters in detail (see Table S2 for illustrative quotes and Table S3 for a list of each cluster’s articles in the supplementary material). Here, it is important to note that neither the cluster names, nor the following detailed descriptions perfectly capture the approaches or emphasis of every article in a given cluster. Rather, they provide general characteristics of the different sustainability-focused narratives that have emerged within the scientific literature on Golden Rice.

**Table 3:** Indicator analysis demonstrated which aspects constituted the character of a certain cluster (no values for the ‘technical effectiveness’ cluster)

Economic efficiency			Equity and holism			Advocacy		
Indicator	Indicator value	*p* val	Indicator	Indicator value	*p* val	Indicator	Indicator value	*p* val
Message: optimistic	0.50	0.001	Culture: needs analysis	0.59	0.001	Message: passionate	0.90	0.001
Project: marketing campaigns	0.47	0.001	Biodiversity: loss	0.57	0.001	Governance: overregulation	0.60	0.001
Cost-utility-analysis	0.39	0.006	Systems thinking and holism	0.55	0.001	Governance: regul. are hurdles	0.54	0.001
Resilience: external influences	0.38	0.003	Values: biases of scientists	0.55	0.001	Resilience: risks are minimal	0.52	0.001
Culture: acceptance	0.37	0.002	Culture: context and habits	0.50	0.001	Power: GMO opposition	0.41	0.001
Power: CIGAR and Rockefeller	0.37	0.007	Equity: inequality	0.46	0.002	Values: against precaut. principle	0.37	0.003
Culture: local data used	0.32	0.005	Philosophy: role of science	0.40	0.002	Resilience: irrational behavior	0.31	0.007
			Message: opposing	0.36	0.003			
			Values: framework proposed	0.34	0.003			
			Power: corporations	0.34	0.014			
			Resilience: complexity	0.33	0.010			
			Resilience: diverse strategy	0.32	0.022			
			Particip.: call for engagement	0.31	0.002			
			Resilience: risks are a concern	0.30	0.003			
			Philosophy: quick fix	0.29	0.008			
			Message: doubtful	0.29	0.001			
			Project: lack of details	0.29	0.004			
			Values: broad consideration	0.28	0.008			
			Participation: local interests	0.25	0.016			
			Equity: empowerment	0.25	0.015			
			Governance: broad consid.	0.23	0.015			
			Equity: negative effects	0.21	0.008			
			Water and soil: altern. farming	0.21	0.012			
			Message: cautious	0.18	0.031			

For detailed information on each abbreviated indicator corresponding to one of our 70 sub-themes see Table S1 in the supplementary material

### Biotechnological branch (*n* = 40)

Articles in the biotechnological branch (*n* = 40) were predominantly authored by plant scientists (65%) and economists (15%); only one paper was written by a social scientist. All held a positive attitude towards Golden Rice, except one with a narrow focus on the potential dangers of beta-carotene engineered plants (Schubert 2008). The overall approach of articles in this branch was to present Golden Rice as an engineering solution to vitamin A deficiency and to argue for broad scale usage of Golden Rice based on measurements of its efficacy in producing beta-carotene. Within the biotechnological branch there were distinct clusters of articles focusing on ‘technical effectiveness’ (red cluster; Fig. 1) and ‘advocacy’ (black cluster; Fig. 1).

#### Technical effectiveness (*n* = 23)

“Another exciting field of modern plant biotechnology is represented by the enhancement of crop nutritional properties through genetic modification [ref.]. There are multiple nutritional advances underway and this review focuses on two representative examples that illustrate the potential impact of this technology.” (Schwember 2008)

As in this quote, articles primarily focused on Golden Rice as an exciting achievement in the reduction of vitamin A deficiency through genetic engineering. Topics addressed in these articles included general overviews of biofortification or GM crops (nine articles), narrow foci on biotechnical processes and resulting effectiveness (six articles), the management of the Golden Rice project itself (five articles) and economic valuation methods for assessing the benefits of GMOs (three articles). Indicator analysis did not result in any predictive sustainability sub-themes within the cluster (no significant indicator values; Table 3). Articles in this cluster generally stated the effectiveness of Golden Rice in regard to the target of producing beta-carotene in the rice endosperm and concluded it would increase the vitamin A status of populations at risk. Considerations of issues such as changes of diets, livelihood strategies or politics were rare.

#### Advocacy (*n* = 17)

“The consequence [of GMO opposition]: millions of avoidable blind and dead children. The author considers those who are responsible for this avoidable suffering of many innocent children (and mothers at childbirth) a crime to humanity […]. There is a wealth of scientific information and broad consensus that GMO-technology is at least as safe as any other technology involved in any context with our food or our environment […]. Our ‘enlightenment’ and science-based successful European culture is on the verge of being replaced by unreason-based failure and lack of culture.” (Potrykus 2013)

As the quote reveals, the tone in this cluster was often extremely emotive, including one author who called the delay of Golden Rice’s implementation a “silent holocaust” (Chassy 2010: 543). This cluster focused on the consequences of regulation GM crops and delayed release of Golden Rice. The unconditional safety of Golden Rice was often stated, invocations of the precautionary principle—that, in the absence of scientific consensus, there is burden of proof for proponents of new products or policies to show that such products or policies are not harmful to humans or the environment (O’Riordan 1994)—were argued as unjustified, and the behaviour of those opposed to agricultural GMOs often framed as irrational. Articles argued for Golden Rice’s implementation with reference to preventable deaths, while not mentioning any concerns related to Golden Rice. The prevailing argumentation built upon the notion of a consensus on Golden Rice’s effectiveness and the absence of any GMO-related risks. The sub-theme ‘message: passionate’ was strongly associated with this cluster in the indicator analysis (indicator value 0.90; Table 3).

Ten articles within this cluster were authored by members of the Golden Rice Humanitarian Board, one by an employee of Monsanto and three other authors have in the past written articles on Golden Rice with members of the board or Monsanto/ Syngenta (totalling in 14 out of 17 articles).^3^

### Socio-systemic branch (*n* = 24)

In comparison to the biotechnological branch, the socio-systemic branch focused less on technical achievements than on the socio-political components/contexts of food systems and vitamin A deficiency. The authors were social scientists (14 articles), economists (6 articles), biologists (2 articles) and interdisciplinary teams (2 articles). Within the branch there were two clusters: ‘economic efficiency’ (blue cluster; Fig. 1) and ‘equity and holism’ (green cluster; Fig. 1).

#### Economic efficiency (*n* = 10)

“We develop a methodology for comprehensive ex ante evaluation […]. We use a truly interdisciplinary approach, integrating epidemiological and nutrition details, as well as socioeconomic and policy factors. In particular, we determine the current public disease burden of VAD in a country with an important rice-eating population, and simulate to what extent this burden could be reduced through GR […]. Finally, we assess the cost-effectiveness of GR…” (Stein et al. 2008)

The commonality of the ‘economic efficiency’ cluster was the focus on cost-benefit calculations, taking into account a wide range of variables, including political and cultural influences. Sixty percent of the articles we authored by economists. Moreover, the significance of sub-themes such as ‘acceptance’ and ‘marketing campaigns’ (Table 3) demonstrated a consumer oriented perspective. In comparison to the clusters in the biotechnological branch, articles in this cluster used multi-factor models in their assessment of Golden Rice and were more likely to consider alternative interventions for addressing vitamin A deficiency (e.g., Zimmermann and Qaim 2004; Stein et al. 2008). Articles tended to give a positive appraisal of Golden Rice, because it was said to be compatible with the current food system and, therefore, more realistic to implement than other interventions, especially due to its cost-effectiveness (e.g., Stein et al. 2008).

#### Equity and holism (*n* = 14)

“Addressing the most immediate and fundamental problems of food insecurity and undernutrition such as micronutrient deficiency, while essential, can only succeed in the long run by proceeding in balance with environmental, sociocultural, political, economic, behavioral and biomedical perspectives.” (Johns and Eyzaguirre 2007)

The ‘equity and holism’ cluster took into account a variety of themes such as participation, equity, biodiversity, water and soil conservation, resilience and system thinking, values and philosophy. The number of statistically significant sub-themes in the indicator analysis exceeded that of other clusters, demonstrating the diversity of the issues and topics that defined this cluster (Table 3). In contrast to other clusters, only one article was in favour of Golden Rice, ascribing it the potential to “play the positive role of technological fixes […]—providing policy-makers with more options and additional means for addressing social problems” (Scott 2011: 225). There was a particular focus on delineating and defining societal goals relating to or intersecting with the potential use of Golden Rice as an intervention for addressing vitamin A deficiency. In line with this prioritization of societal goals, most articles raised concerns over Golden Rice’s adequateness as solution to vitamin A deficiency (e.g., Lorch 2001; Nestle 2001; Small 2014). Articles called into question Golden Rice’s ‘real world’ nutritious efficacy in relation to contextual factors such as diet, the presence of other infectious diseases (Egana 2003) or storage losses (Stone and Glover 2016). There was also consideration of environmental and social consequences of a continued reliance on ‘mega-crops’ (Small 2014), the ‘placelessness’ of Golden Rice and a lack of transferability to local food systems (Stone and Glover 2016) as well as the disregard of indigenous knowledge (Johns and Eyzaguirre 2007). These concerns highlighted the need to consider complex preconditions or confounding factors in the successful use of Golden Rice as a mitigation strategy, rather than seeking to dismiss the benefits of Golden Rice itself.

### Representation of sustainability themes

While the focus on sustainability themes and sub-themes varied widely between clusters (Table 3), there were certain sub-themes that received less attention than others. Issues that were addressed in less than 15% of the reviewed articles (largely the socio-economic branch) concerned: local interests, life quality, dignity, empowerment, autonomy, poverty alleviation, climate change, alternative farming methods, systems thinking, system dynamics across scales, details on Golden Rice’s distribution and on its monitoring, trade policies, governance and ethics. The least addressed issues such as dignity, food sovereignty, alternative farming methods and climate change (five, three, three and two mentions respectively) were largely absent across all four clusters.

## Discussion

The conclusions to be drawn from our results are threefold: (1) the body of literature on Golden Rice can be grouped into clusters whose range of sustainability themes correlated with the articles’ evaluation of Golden Rice and the authors’ discipline; (2) the biotechnological branch represented the dominant narrative in terms of quantity of articles, yet lacked a focus on crucial sustainability themes; and (3) there was little integration or overlap between the thematic foci or broader perspectives of the two branches, and particularly polarized positions arose in the clusters on ‘advocacy’ (e.g., Potrykus 2013) and ‘equity and holism’ (e.g., Small 2014). Such polarized debates are useful for identifying the initial differences in visions, goals and values that shaped these discourses. However, it is vital to understand the sources of such disagreements to move beyond polarization towards dialogue and mutual benefit. To inform and facilitate this process, it is useful to consider what paradigms^4^ underpin the two branches of the literature, and their influences on respective problem framings and narratives surrounding Golden Rice.

### Paradigms underpinning branches

The observed homogenous composition of either social or natural scientists (and their corresponding evaluations of Golden Rice) in each branch suggest the existence of a disciplinary dichotomy. This divide between scientific cultures presents a major challenge to integration (Tress et al. 2005), although it is not the only factor leading to polarized positions. For example, Legwegoh and Fraser (2015) argue opportunism and political economy have led to a similar case of diverging narratives in the context of the food security discourse.

The biotechnological branch’s arguments are built on premises often shared by the natural science community. A core assumption is the existence of an objective reality that can be investigated, described and to a certain extent predicted based on generalized, reductionist theories (Becher 1994; Moon and Blackman 2014). In the case of Golden Rice, this approach helps shape the observed focus on theoretical assessment of the effectiveness of plants in producing vitamin A from a purely biophysical perspective. Following positivist logic, the effectiveness in biophysical terms would translate into a successful mitigation of vitamin A deficiency in the ‘real world’. More profoundly, the idea of engineering a plant to contain beta-carotene might be traced to a framing of the problem that is characteristic to natural sciences. The nature of the problem, that is the cause of malnutrition, was perceived to be related directly to proximate biophysical factors (the lack of beta-carotene in rice plants), and not on less proximate factors such as poverty. This relatively narrowly framed problem definition naturally lends itself to a technical solution, and one aligned to the authors’ own expertise. In contrast, the existence of resource poor farmers was regarded as given and, therefore, not as target of scientific efforts (Scott 2011). Such bounded scientific enquiry results in generic notions of effectiveness, suggesting universal applicability directed at “the poor” (e.g., Chassy 2010) as a homogenous group, in “developing countries” (e.g., Zimmermann and Qaim 2004)—an unspecified global space (Stone and Glover 2016).

In the socio-systemic branch the object of research shifted from the natural environment to human behaviour. Principles from the social sciences were applied, such as relativism and intersubjectivity (Moon and Blackman 2014), and informed a perception of the world as a multidimensional and interconnected system, whose variables cannot be understood in isolation (Loos et al. 2014). As result, the research centred on power and justice, systems thinking, participation and ethics within a specific place-based case, as typified by the ‘equity and holism’ cluster (Fig. 1; Table 3).

The ‘economic efficiency’ cluster only partially fits within this characterization, because most economists carry a distinct set of assumptions, rooted in rationalistic-individualistic, neoclassical, utilitarian paradigms (Becher and Trowler 2001; Etzioni 2010). These assumptions tended to favour models for assessing Golden Rice’s cost-effectiveness based on explicit assumptions informed by the study of the causal links between systems components. Quantitative methods and generalized models prevailed in this branch. This focus on generalizable models, as legitimate approaches for addressing context dependent real world problems, provides a clear link with biotechnological branch of Golden Rice research, despite the considerable differences in thematic foci between the biotechnological and socio-systemic approaches.

### Distinct strengths as impetus for integration

The two branches of literature presented different strengths in their approaches to conceptualizing and solving the problem of vitamin A deficiency. The biotechnological branch offered a focused, generalizable, quickly transferable, one-time intervention, effective in regard to its target—the provision of a beta-carotene producing rice variety (e.g., Potrykus 2001). This rice might act as positive example of a technological fix as suggested by Scott (2011). However, the lack of focus on confounding factors that are likely to influence the success of Golden Rice, limits the nuanced understanding of how such broad brush interventions will play out in what are inevitably complex, context specific socio-political contexts.

In contrast, the socio-systemic branch’s broader, more contextualized understanding of vitamin A deficiency promoted strategies embracing the traditional approaches of supplementation and fortification along with capacity building in agroecology and education on nutrition, hygiene and health. This approach aims at synergies (e.g., by combing health checks with education measures) and at a broad notion of well-being, taking into account various aspects of health and sustainable livelihood strategies, not just vitamin A deficiency (e.g., Johns and Eyzaguirre 2007). Such contextual approaches face major barriers though, often not being compatible with the status quo of a targeted food system or current politics (Scoones 2002; Scott 2011). Moreover, the strong focus on the importance of context in sustainability problem framing may diminish the potential positive contribution that general, broad-brush technical solutions can have if contextual issues are addressed.

A constructive dialogue on strengths and limitations of both approaches might serve to draw a more nuanced picture of Golden Rice and to eventually inform better research outcomes by aligning both technical depth (e.g., how to design optimal seeds and growing conditions) and thematical breadth (e.g., which components of the food system influence vitamin A deficiency to what extent).

### Values and vested interests in science

Despite the high promises of cooperation between the socio-systemic and the biotechnological branch, our results indicated profound differences in their respective problem framings. These problem framings required different research methods and team constellations and concluded in diverging solutions, working on different scales and time frames (short term changes in plant metabolism versus long term food system transformation). It is necessary not just to contend with the way different disciplinary traditions shape sustainability problem framing, but also with diverging values and worldviews among researchers personally (Garnett 2013). Moreover, there is a need to address the feedbacks between personal values and disciplinary traditions. Personal values influence individuals’ choice of discipline, and those—self-selecting—scholarly communities tend to reinforce particular worldviews. Nevertheless, the disciplinary divide seems inadequate as an explanation for the high extent of polarization between the clusters within the academic literature. Are GM crops safe, beneficial to biodiversity and a key to food security? Is it legitimate to base GMO regulations on the precautionary principle? Is our current agricultural system in crisis or at a historical peak? Polarized positions on these questions regardless of affiliations point to the influence of values and deeply held worldviews on framing the research (Fischer et al. 2014) and on interpreting results (Devos et al. 2014). These normative propositions of researchers were often obscured in the Golden Rice discourse by the assumed objectivity of scientific research. Accordingly, authors often made dichotomous policy recommendations, either supporting or rejecting Golden Rice and thus portraying the case as a formal objective problem, solvable within the realm of deduction (Levidow and Marris 2001; Herrick 2004), rather than as a normative and value-laden issue.

Moreover, in some instances vested interests have led scientists to ally with corporate representatives or activists, thereby increasing the divergence between positions through the self-amplifying process of “social bonding against a common enemy” (Stone 2017: 590). Despite the strong influences of disciplinary affiliations, profound ideological divides, and entanglements between science and policy, our analysis showed little recognition of how these topics shape and polarize the Golden Rice discourse.

### Pathways to sustainability

Offering a sustainability science perspective on how to move the debate forward, we suggest a reframing of the question and its research methodology, by prioritizing human well-being and local involvement. To transcend the reductionism of regarding rice as mere nutrient provider, neglecting its place in the eco- and cultural system (Hayes-Conroy and Sweet 2014), and of describing vitamin A-deficient populations as passive victims (‘the poor’) in unspecified geographic and social positions, we propose to reframe the question: from ‘how do we create a rice plant producing beta-carotene?’ or ‘how do we most efficiently raise the vitamin A status of populations at risk?’ to ‘how do we foster the well-being of people affected by malnutrition, both in short and long terms?’. Such a reframing of putting people first automatically aligns health and nutrition with equality, secure livelihoods and environmental integrity (Bennett 2017). Most importantly, to understand what well-being means to the people in question, there is no way around asking. This necessary physical proximity creates room for participation, for joint agenda setting, for mutual learning, for producing ‘socially-robust’ knowledge (Gibbons 1999), in short: for the aims and rationales of transdisciplinarity (Lang et al. 2012).^5^

Indeed participation of non-scientists in both problem framing and solution formation was largely overlooked in the Golden Rice literature, with a notable lack of focus on sub-themes such as local culture, participation, dignity and empowerment in the articles reviewed here. This lack of participation exists despite the obstructive mistrust towards Golden Rice, witnessed both in the Global North (Baggott 2006) and South (Cabanilla 2007). Despite challenges in praxis (Brandt et al. 2013; Kenny et al. 2015) and a limited number of evaluation studies (Bath and Wakerman 2015), there is a growing recognition of the feasibility and potential success of transdisciplinarity, for example, in the field of health policy and systems research (Sheikh et al. 2014) or agricultural research (Hoffmann et al. 2007; Neef and Neubert 2011). Such approaches potentially allow for socio-technical solutions that can be adapted to specific socio-political or socio-ecological contexts and that acknowledge that multiple interventions are often required to fix what at first glance might seem like relatively simple problems (such as a vitamin A-deficient diets).

More broadly, polarized discourses regarding solutions to pressing sustainability problems may be avoided, or at least diminished, by attempts to develop shared problem definitions (both across different scientific disciplines and in conjunction with those who are impacted by the proposed solutions). This requires greater focus on exploring the way sustainability science is shaped by disciplinary traditions, underpinning assumptions, values and world-views. Furthermore, we argue that the seeming adversarial perspectives on sustainability problems that arise from more technical or socio-political perspectives may actually be complementary. The development of socio-technical solutions that seek to bridge the divide between overgeneralized technical solutions and deeply contextualized socio-political approaches with limited transferability would increase the applicability and legitimacy of sustainability science. For this to occur rather than competing narratives developed in tandem, what is required is genuine dialogue that acknowledges the underpinning factors (including problem framing) that can lead to such fractured discourses in sustainability science. Dialogue and mutual understanding denote a starting point for deeper-level institutional changes that are necessary to facilitate and mainstream inter- and transdisciplinary research projects. Currently existing institutions in science pose structural constraints to greater inclusivity. These institutional constraints include reward mechanisms that incentivise specialisation and a lack of regard for specific outputs of inter- and transdisciplinary research such as knowledge co-creation (National Research Council 2004; Schneidewind 2010). As Ostrom (1990) described, changes must happen not only at the level of operational and collective rule-making, but also at the constitutional level—in this case, the organising principles and power relationships of the institutions of science.

## Conclusion

The findings of normative science, including in sustainability science, can be highly polarized (e.g., Fischer et al. 2014). Using the Golden Rice as an exemplar of such polarized debates we found that the obstacles to integration of knowledge arose from diverging problem framings, here explained as connected to disciplinary affiliation and personal values. To overcome these obstacles to shared understanding we proposed three steps: (1) to explicitly recognize why a situation is framed as a problem and what criteria constitute sustainability in the particular context; (2) to work in transdisciplinary ways, based on mutual respect, by prioritizing well-being and systems thinking; and (3) to reflect on the potentials and limitations of academia’s current institutions in facilitating inter- and transdisciplinarity. These steps may facilitate the overall aim of addressing root causes of unsustainability in, by and through science.

## Electronic supplementary material

Below is the link to the electronic supplementary material.

Supplementary material 1 (DOCX 57 KB)

## Footnotes

1 In comparison, raw sweet potato contains 85.1 µg/g beta-carotene (USDA 2016). It is the only of the 10 most produced staple crops exceeding second generation Golden Rice (FAOSTAT 2016).

2 Because economists tend to have a very specific approach to the normative issues addressed here, we classified them separately from other social scientists.

3 Technically seen, Monsanto is not involved in the project, having donated patents for a gene that has already been replaced, yet Stone and Glover stated the company “has been eager to take credit for Golden Rice” (2016). Further authors ascribed Golden Rice a marketing value as it facilitates consumer acceptance and eventual market penetration of Syngenta and Monsanto (Nestle 2001; Brooks 2011). Thus, we conclude the Golden Rice Humanitarian Board and biotech corporations share the interest of bringing Golden Rice to the fields.

4 The notion of paradigms is used to describe “a set of assumptions that structure the approach to research” (Moon and Blackman 2014: 1173).

5 Following Lang et al. (2012), we define transdisciplinary as scientific principle of knowledge co-creation in teams of different disciplines and actor groups targeted at mitigating societal problems.
