# Supplementary material for: From disagreements to dialogue: unpacking the Golden Rice debate
Source: Sustain Sci. 2018 May 17;13(5):1469–82. doi: 10.1007/s11625-018-0577-y (PMC6132390; doi:10.1007/s11625-018-0577-y)
Supplement: Supplementary file 1 — Supplementary material 1 (DOCX 57 KB) [file 11625_2018_577_MOESM1_ESM.docx]

# Supplementary Material

## Table S1. List of 70 sub-themes informing quantitative analysis, based on themes derived from sustainability literature (Table 1), GR: Golden Rice, VAD: vitamin A deficiency.

**Message**

- Neutral: no judgement
- Cautious: a lack of empirical data prohibits judgement
- Doubtful: GR is regarded as an option, but serious concerns are mentioned and/or more promising solutions are proposed
- Opposing: rejection of GR due to its described inadequateness
- Optimistic: GR is a promising solution to VAD, there are minor reservations
- Passionate: if GR will not be approved fast children die, no concerns expressed

**Personal Background**

- Mixed Team
- Global South
- Global North

**Affiliation**

- Interdisciplinary
- Social Science incl. Philosophy
- Economics
- Nutrition
- Plant Science
- Others
- Employee of Syngenta or Monsanto
- Part of the GR Humanitarian Board or working at one of the network’s institutes on GR

*Does the respective article address…?*

**Participation**

1. Does it call for an engagement of stakeholders in the choice of a malnutrition strategy/ of GR’s regulation?
2. Does it name local interests?
3. Have interviews been conducted?

**Local culture**

1. Has local data been used?
2. Does it analyze the need for GR or speak of such analysis as prerequisite? (Only coded if the argument for GR’s necessity goes beyond “rice does not contain beta-carotene”)
3. Does it address the acceptance of farmers and consumers?
4. Does it speak of eating habits (beyond a large share of rice in diet) and/or provide context information, name social norms?

**Health & well-being**

1. Does it name predictors/causes of VAD? Min. 3 out of: poverty; lack of access to land; parasitic infestations; seasonality; education; refugees/famine; life stage; eating habits: polished rice, food variety, vegetarianism, fat in diet, breast-feeding
2. Does it list consequences of VAD?
3. Does it explain the interrelations of deficiencies, parasites and diseases, either as mutual reinforcement or the effect of other diseases on VAD? (Only naming a weakened immune system is regarded as consequence.)
4. Does it mention well-being or life quality?

**Dignity & human rights**

1. Does it speak of human rights or dignity?
2. Does it address autonomy and self-determination of local people, in the sense of food sovereignty?

**Equity & empowerment**

1. Does it mention inequality and social disparities or distributional justice?
2. Does it speak of empowerment & education?
3. Does it state a positive effect of GR on poverty & access to food (e.g. commercial advantage for GR farmers, more productive workforce)?
4. Does it state a negative effect of GR on poverty (e.g. competing for resources in poverty alleviation) or no change of situation?

**Actors, resources & power**

1. Does it speak of power relations, networks or dynamics?
2. Does it name media as relevant actor?
3. Does it name the GMO opposition as relevant actor?
4. Does it describe the role/interest of corporations?
5. Does it describe the role/interest of scientists?
6. Does it name national governments as relevant actors?
7. Does it name the CIGAR network or philanthropic foundations as actor?
8. Does it ascribe GR a potential as marketing tool or as business opportunity?

**Governance & institutions**

1. Does it speak of current biotech regulation policies as hurdle?
2. Does it speak of current biotech regulation policies as illegitimate over-regulation?
3. Does it speak of biotech regulation in a neutral tone or describe them as a prerequisite for safe use?
4. Does it speak of property rights?
5. Does it speak of international institutions and unions (UN, WHO, WTO, EU…)?
6. Does it speak of local governance concerning the regulation and monitoring in target countries?
7. Does it address governance issues on a broader level?
8. Does it name effects of international trade policies?

**Climate change**

1. Does it mention climate change?

**Biodiversity**

1. Does it speak of a potential loss of biodiversity (through monocultures, dominance of few cultivars or outcrossing)?
2. Does it state there will be no adverse effect on biodiversity (compatible with traditional landraces)?

**Water and soil conservation**

1. Does it speak of current adverse effects of industrial agriculture on soil and water?
2. Does it propose alternative agricultural practices like agroecology?
3. Does it state GR will have no adverse environmental impact or even a positive one?

**Resilience & risks**

1. Does it describe the issue as complex and/or uncertain?
2. Does it state that case-by-case assessments are needed and/or that risks are a concern?
3. Does it state that risks are negligible or non-existent?
4. Does it propose a diversified strategy to combat malnutrition?
5. Does it propose long term poverty alleviation?
6. Does it propose GR to be a complement only to fortification & supplementation?
7. Does it describe GR as superior to other strategies?
8. Does it describe external influences on the system, esp. threats and/or GR’s possible reaction to those?
9. Does it speak of emotions and irrational behavior?

**Holism & systems thinking**

1. Does it call for holism and/or speak in systems thinking terminology?
2. Does it make use of modelling, diagrams and/or explicit explain causal relations and underlying assumptions?
3. Does it describe system dynamics across scales?

**Cost-utility-analysis**

1. Does it weigh cost and utility of GR’s implementation?
2. Does it distinguish short and long-term effects of GR or of biofortification in general?

**Achievability & realization prospects**

1. Does it reflect on the feasibility of bringing GR to the consumer?
2. Does it speak of an evaluation/ monitoring of GR following implementation or of royalty payments of farmers earning more than $10,000?
3. Does it name cost estimations of parts of the implementation process or the entire GR project?
4. Does it address the nutritious efficacy of GR?
5. Does it criticize a lack of details on GR’s rollout?
6. Does it comment on the funding of the distribution?
7. Does it name concrete project steps?
8. Does it mention information campaigns as tool to increase adoption?

**Values & transparency**

1. Does it propose a framework to evaluate GR?
2. Does it mention biases of scientists and/or advocacy?
3. Does it regard the global spread of biotechnology as moral imperative?
4. Does it describe ethics, principles and/or heuristics in a broad sense?
5. Does it describe the precautionary principle as useful?
6. Does it describe the precautionary principle as unnecessary and hindrance to innovation?
7. Does the author disclose his or her standpoint?

**Philosophy & reflection**

1. Does it address the technological fix criticism?
2. Does it reflect on the role of science/scientists?

## Table S2. Quotes illustrating each cluster’s narrative, five quotes per cluster.

**Technical effectiveness (n=23)**

“This significant achievement in nutritional biofortification of rice endosperm was a major milestone in combating vitamin A deficiency and its associated effects. Golden Rice has now been demonstrated as an effective source of vitamin A [ref.] and studies that focused on Golden Rice in view of public health in India suggest that, with governmental and public support, it has the potential to significantly reduce the disease burden of vitamin A deficiency [ref.].” (Bhullar & Gruissem, 2013, p. 52)

“Genetic modification (GM) technology has provided a solution to overcome this problem [of vitamin A deficiency (VAD)]: rice developed with the help of GM technology at the ETH Zurich and the University of Freiburg, known as Golden Rice (GR), which is claimed to be a source of VA [ref.]. Therefore, to achieve the millennium development goals (MDGs), particularly elimination of VAD, GR is likely to play a significant role. The commercial cultivation of GR is likely to commence in some Asian countries around 2012 [ref.].” (Kajale & Becker, 2014, p. 2)

“Another exciting field of modern plant biotechnology is represented by the enhancement of crop nutritional properties through genetic modification [ref.]. There are multiple nutritional advances underway and this review focuses on two representative examples that illustrate the potential impact of this technology.” (Schwember, 2008, p. 241)

“Poor people whose diets primarily consist of cereal grains and tubers have serious prevalence of VAD. Thus, the focus of crop improvement for enhancing vitamin A is on cereal and tuber crops. The main target for provitamin A biofortification is b-carotene, and increased carotenoid accumulation can be achieved by directing and enhancing flux into carotenoid biosynthetic pathway or by downregulating the turnover of b-carotene [ref.]. The best example of the former strategy is the development of so-called Golden Rice [ref.].” (Khush, Lee, Cho, & Jeon, 2012, p. 198)

“Rice feeds more than half the human population worldwide, most of whom live in developing countries and many have (at least during certain seasons) no other or virtually no other diet. Rice contains small amounts of Fe and provitamin A in the aleurone layer of the seeds, which is polished away in order to prevent the kernels becoming rancid. Consequently, vitamin A and Fe deficiencies are widespread. Although certain rice varieties have been reported to contain more provitamin A or Fe in the central part of the seeds [ref.], traditional breeding has not yet solved the problem. Although for vitamin A the problem is close to being solved, for Fe a solution is currently actively being sought. These studies will be reviewed.” (Sautter, Poletti, Zhang, & Gruissem, 2006, p. 155)

**Advocacy (n=17)**

“The moral calculus is surprisingly simple: if GR had been distributed in 2002 or 2003, millions of lives might have been saved. Not to have disseminated the seeds of GR until now has allowed as many people to die silently as were killed in the holocaust.”(Chassy, 2010, p. 543)

“What else is necessary to open the eyes of those, who carry political responsibility, to understand that not changing GE-regulation from extreme precautionary to science-based regulation, guided by considerations of the risks and benefits of the trait instead of regulating a technology on ideological terms, constitutes a ‘crime against humanity’ [ref]?” (Potrykus, 2010, p. 472)

“The consequence [of GMO opposition]: millions of avoidable blind and dead children. The author considers those who are responsible for this avoidable suffering of many innocent children (and mothers at childbirth) a crime to humanity. There are those who commit this deliberately and those who are participating passively, such as numerous ‘humanitarian organizations’ and ‘decision makers’ in politics and elsewhere. There is a wealth of scientific information and broad consensus that GMO-technology is at least as safe as any other technology involved in any context with our food or our environment. What we experience here is an example of ‘unreason’ and a perfect example in the context of The March of Unreason. Our ‘enlightenment’ and science-based successful European culture is on the verge of being replaced by unreason-based failure and lack of culture.” (Potrykus, 2013, p. S86)

“The controversy over Golden Rice has been very frustrating to many of us. As Potrykus says, it is unfortunate that offering a technology for free that could save so many children and pregnant mothers has been delayed for so long. Since the invention of Golden Rice, 2.5 million children are estimated to have died each year from VAD. Around 500,000 go blind each year, of whom 70 % die. They wouldn’t all have been saved by Golden Rice, of course, but every delay means many unnecessary dead or blind children [ref.]. As IRRI’s director general, I believe that it is high time that activists stop using their cynical and destructive ways to oppose Golden Rice and instead support scientific research, development, and assessment of the product because of its strong potential benefit to humanity.” (Zeigler, 2014, p. 260)

“EU opposition and NGO domino politics are the real reasons we have been waiting since 1982 for the eradication of blindness in the developing world. NGOs like Greenpeace will never reconsider. Publics, however, could be a different story. The most important group to sway is the European public (…). Awareness should be premised on three key facts: GM foods have never been shown harmful. Golden Rice is not intended for export to Europe, and the obstructionism of their governments is allowing the blindness and deaths of hundreds of thousands. Public awareness campaigns like those conducted by the Golden Rice Project, as well as the addition of the topic to the local Rotary Club meeting, could do much to promote grassroots knowledge. Additionally, the United States should try to get the European Union and developing countries talking specifically about Golden Rice, not the far more threatening specter of GM foods at large.” (Baggott, 2006, p. 30)

**Economic efficiency (n=10)**

“We develop a methodology for comprehensive ex ante evaluation, which substantially improves upon the previous, more partial impact studies. Dawe et al. (2002) focused on the potential effects of GR on b-carotene intakes, but without considering actual health impacts. Zimmermann and Qaim (2004) considered health aspects, but only at a highly aggregate level and without taking into account important nutritional features like dietary heterogeneity across different regions and social groups or the role of reference intakes in dietary assessments. We use a truly interdisciplinary approach, integrating epidemiological and nutrition details, as well as socioeconomic and policy factors. In particular, we determine the current public disease burden of VAD in a country with an important rice-eating population, and simulate to what extent this burden could be reduced through GR. The simulations build on new insights of the technology’s efficacy [ref]. Finally, we assess the cost-effectiveness of GR more comprehensively than the previous work and compare the results with the cost-effectiveness of alternative VA interventions and other public health programs.” (Stein, Sachdev, & Qaim, 2008, p. 145)

“The larger moral point is that the perfect should not be the enemy of the good; in general, even if the best ethical analysis of existing systems demonstrates that they are seriously flawed, it would not without further argument justify a categorical refusal to improve those systems, even if ideally a more comprehensive, but also more remote, solution be to replace them entirely with better ones.” (Hessler, 2011, p. 275)

“In this context, widespread consumption of Golden Rice could reduce the burden by 59%, which includes the saving of almost 40,000 lives every year (Table 3). Because the severity of VAD is negatively correlated with income, the positive effects are most pronounced in the poorest income groups (Stein et al., 2008). While these results suggest that Golden Rice alone is unlikely to eliminate the problems of VAD, the projected improvements in public health and nutrition are huge.” (Qaim, 2011, p. 45f.)

“Their influence [of NGOs advocating organic farming] is not to be ignored, and if GR is to be adopted, educational campaigns targeted to farmers and the general public will be of crucial importance. There are several other risks that could be important. First, after substantial investment, GR may not be widely adopted and will have little semblance of the impact envisioned. Farmers who wish to sell it in markets (…) may not want to take the risks of adopting a new variety (…) unless they are compensated with higher prices or yields.” (Dawe & Unnevehr, 2007, p. 158)

“This paper analyses potential impacts in a Philippine context. Since the technology is still at the stage of R&D, benefits are simulated with a scenario approach. Health effects are quantified using the methodology of disability-adjusted life years (DALYs). Golden Rice will not completely eliminate the problems of vitamin A deficiency, such as blindness or increased mortality. Therefore, it should be seen as a complement rather than a substitute for alternative micronutrient interventions. Yet the technology could bring about significant benefits. Depending on the underlying assumptions, annual health improvements are worth between US$ 16 and 88 million, and rates of return on R&D investments range between 66% and 133%. Due to the uncertainty related to key parameters, these results should be treated as preliminary.” (Zimmermann & Qaim, 2004, p. 147)

**Equity & holism (n=14)**

“Unless participatory approaches are used to reflect indigenous knowledge and cultural systems and are gender sensitive with respect to women’s knowledge, contributions and needs, biofortification may not succeed as intended. (…) Addressing the most immediate and fundamental problems of food insecurity and undernutrition such as micronutrient deficiency, while essential, can only succeed in the long run by proceeding in balance with environmental, sociocultural, political, economic, behavioral and biomedical perspectives. (…). Focusing on staple cereals is unlikely to benefit the poor for economic and nutritional reasons alike, and as it leads to even greater dietary simplification can do more harm than good. Biofortification would better focus on local vegetatively propagated species.” (Johns & Eyzaguirre, 2007, p. 17ff.)

“Golden rice will not offer the solutions needed for the elimination of VAD/xerophthalmia and indeed the researchers themselves know this. There were several instances where golden rice as an intervention appears to fall short of providing the holistic approach needed to eliminate VAD/xerophthalmia. For instance, the prevalence of parasitic infestation and inadequate fat intake in high-risk communities have to be taken into account if real breakthroughs are to be made.” (Egana, 2003, p. 174)

“While those involved in the project praise it as a new form of private-public partnership in a humanitarian setting, critics consider it an inappropriate high-tech solution to the complex problem of food access, which has been taken up by life sciences companies and used for their own public relations. Ultimately, however, it will be governments, development organizations and local people who will have to make the choice about the measures they decide to adopt to combat malnutrition.” (Lorch, 2001, p. 22)

“Until Western dietary practices were introduced, indigenous people historically avoided vitamin A deficiency by consuming local green leafy vegetables. Engelberger carried out a search for abandoned native provitamin-A-rich plants. These were identified, and proved to provide a much more reasonable solution than the ‘vitamin A fiasco’ [ref.] supplementation programme previously implemented. Bruinsma [ref.] noted that ‘Golden Rice could have counterproductive impacts on nutritional problems by curtailing the progress made in educating people to diversify their diet and increase the diversification of agriculture production’. Indeed, there is a disconcertingly imperialistic aspect about the efforts of affluent interests to thrust ‘biotechnology for the poor’ on the Global South [ref.].” (Small, 2014, p. 285)

“The practical criticisms alert one to the ambiguity of technological fixes. They can be said to solve problems only when the criteria for success is narrowly defined in the manner of an engineering problem. (…) It [Golden Rice] could play the positive role of technological fixes as listed by Weinberg – providing policy makers with more options and additional means for addressing social problems. The debate over biotechnology is in part a debate over how to frame the challenges of twenty-first century agriculture. It is obvious that these challenges are scientific and technological as well as social and political.” (Scott, 2011, p. 225)

## Table S3. List of 64 articles included in analysis and their respective cluster.

**Biotechnological branch (n=40)**

*Technical effectiveness (n=23)*

Banta LM, Montenegro M (2008) Agrobacterium and plant biotechnology. Agrobacterium From Biol to Biotechnol 73–147. doi: 10.1007/978-0-387-72290-0_3

Beyer P (2010) Golden Rice and “Golden” crops for human nutrition. N Biotechnol 27:478–481. doi: 10.1016/j.nbt.2010.05.010

Bhullar NK, Gruissem W (2013) Nutritional enhancement of rice for human health: The contribution of biotechnology. Biotechnol Adv 31:50–57. doi: 10.1016/j.biotechadv.2012.02.001

Chikkappa GK, Tyagi NK, Venkatesh K, et al (2011) Analysis of transgene(s) (psy+crtI) inheritance and its stability over generations in the genetic background of indica rice cultivar Swarna. J Plant Biochem Biotechnol 20:29–39. doi: 10.1007/s13562-010-0021-6

Corrigan JR, Depositario DPT, Nayga RM, et al (2009) Comparing open-ended choice experiments and experimental auctions: An application to golden rice. Am J Agric Econ 91:837–853. doi: 10.1111/j.1467-8276.2009.01267.x

Datta SK, Datta K, Parkhi V, et al (2007) Golden rice: Introgression, breeding, and field evaluation. Euphytica 154:271–278. doi: 10.1007/s10681-006-9311-4

Depositario DPT, Nayga RM, Wu, Jr. X, Laude TP (2009) Effects of Information on Consumers’ Willingness to Pay for Golden Rice. Asian Econ J 23:457–476. doi: 10.2202/1542-0485.1058

Diretto G, Fiore A, Giuliano G (2007) Metabolic engineering of carotenoid levels for improvement of plants as food. CAB Rev Perspect Agric Vet Sci Nutr Nat Resour. doi: 10.1079/PAVSNNR20072039

Glenn KC (2008) Nutritional and Safety Assessments of Foods and Feeds Nutritionally Improved through Biotechnology : Case Studies Prepared by a Task Force of the ILSI International Food Biotechnology Committee. Asia Pac J Clin Nutr 17:37–104. doi: 10.1111/j.1750-3841.2007.00579.x

ILSI International Food Biotechnology Committee (2008) Golden Rice 2. Compr Rev Food Sci Food Saf 7:92–98. doi: 10.1111/j.1541-4337.2007.00029_7.x

Kajale DB, Becker TC (2014) Willingness to Pay for Golden Rice in India: A Contingent Valuation Method Analysis. J Food Prod Mark 21:319–336. doi: 10.1080/10454446.2012.726946

Khush GS, Lee S, Cho J Il, Jeon JS (2012) Biofortification of crops for reducing malnutrition. Plant Biotechnol Rep 6:195–202. doi: 10.1007/s11816-012-0216-5

Moghissi a A, Pei S, Liu Y (2015) Golden rice: scientific, regulatory and public information processes of a genetically modified organism. Crit Rev Biotechnol 0:1–7. doi: 10.3109/07388551.2014.993586

Najafi B. H. MBH ;Le. (2014) Biotechnology and its impact on food security and safety. Curr Nutr Food Sci 10:94–99. doi: http://dx.doi.org/10.2174/1573401310666140306225243

Paine JA, Shipton CA, Chaggar S, et al (2005) Improving the nutritional value of Golden Rice through increased pro-vitamin A content. Nat Biotechnol 23:482–7. doi: 10.1038/nbt1082

Potrykus I (2010) The private sector’s role in public sector genetically engineered crop projects. N Biotechnol 27:578–581. doi: 10.1016/j.nbt.2010.07.006

Sautter C, Poletti S, Zhang P, Gruissem W (2006) Biofortification of essential nutritional compounds and trace elements in rice and cassava. Proc Nutr Soc 65:153–159. doi: 10.1079/PNS2006488

Schubert DR (2008) The problem with nutritionally enhanced plants. J Med Food 11:601–605. doi: 10.1089/jmf.2008.0094

Schwember AR (2008) An update on genetically modified crops. Cienc e Investig Agrar 35:231–250.

Stein AJ, Sachdev HPS, Qaim M (2006) Potential impact and cost-effectiveness of Golden Rice. Nat Biotechnol 24:1200–1201. doi: 10.1038/nbt1006-1200b

Tang G, Jian Q, Dolnikowski GG, et al (2009a) Golden rice is an effective source of vitamin A. Am J Clin Nutr 89:1776–1783. doi: 10.3945/ajcn.2008.27119

Tang G, Russell RM, Qin J, et al (2009b) Reply to MB Krawinkel. Am J Clin Nutr 90:696–697. doi: 10.3945/ajcn.2009.28268

Yonekura-Sakakibara K, Saito K (2006) Review: Genetically modified plants for the promotion of human health. Biotechnol Lett 28:1983–1991. doi: 10.1007/s10529-006-9194-4

*Advocacy (n=17)*

Al-Babili, S., Beyer, P., 2005. Golden Rice - Five years on the road - Five years to go? Trends Plant Sci. 10, 565–573. doi:10.1016/j.tplants.2005.10.006

Anderson, K., Jackson, L.A., 2005. Some implications of GM food technology policies for Sub-Saharan Africa. J. Afr. Econ. 14, 385–410. doi:10.1093/jae/eji013

Baggott, E., 2006. A Wealth Deferred. Harvard Int. Rev. 28, 28.

Chassy, B.M., 2010. Food safety risks and consumer health. N. Biotechnol. 27, 534–544. doi:10.1016/j.nbt.2010.05.018

Dubock, A., 2014. The politics of Golden Rice. GM Crops Food 5, 210–22. doi:10.4161/21645698.2014.967570

Eisenstein, M., 2014. Biotechnology: Against the grain. Nature 514, S55–S57. doi:10.1038/514S55a

Mayer, J.E., 2005. The Golden Rice Controversy: Useless Science or Unfounded Criticism? Bioscience 55, 726. doi:10.1641/0006-3568(2005)055[0726:TGRCUS]2.0.CO;2

Mayer, J.E., Potrykus, I., 2011. Golden rice’ and biofortification - Their potential to save lives is being hampered by overzealous regulation. Acta Hortic. 941, 21–34.

Potrykus, I., 2013. Genetic Modification and the Public Good. Eur. Rev. 21, S68–S79. doi:10.1017/S 1062798713000203, Published online: 23 July 2013

Potrykus, I., 2010. Lessons from the “Humanitarian Golden Rice” project: Regulation prevents development of public good genetically engineered crop products. N. Biotechnol. 27, 466–472. doi:10.1016/j.nbt.2010.07.012

Potrykus, I., 2008. Is GMO Over-regulation Costing Lives?, in: Health for All?: Analyses and Recommendations. pp. 59–74. doi:10.1002/9783527619511.ch14

Potrykus, I., 2001. The Golden Rice “Tale.” Vitr. Cell. Dev. Biol. - Plant 37, 93–100. doi:10.1079/IVP2000168

Qaim, M., 2010. Benefits of genetically modified crops for the poor: Household income, nutrition, and health. N. Biotechnol. 27, 552–557. doi:10.1016/j.nbt.2010.07.009

Sherman, J.H., Choudhuri, S., Vicini, J.L., 2015. Transgenic proteins in agricultural biotechnology: The toxicology forum 40th annual summer meeting. Regul. Toxicol. Pharmacol. 73, 811–818. doi:10.1016/j.yrtph.2015.10.014

Wesseler, J., Zilberman, D., 2014. The economic power of the Golden Rice opposition. Environ. Dev. Econ. 19, 724–742. doi:10.1017/S1355770X1300065X

Zeigler, R.S., 2014. Biofortification: Vitamin A Deficiency and the Case for Golden Rice, in: Plant Biotechnology. Springer International Publishing, Cham, pp. 245–262. doi:10.1007/978-3-319-06892-3_19

Zilberman, D., Kaplan, S., Wesseler, J., 2015. The loss from underutilizing GM technologies. AgBioForum 18, 312–319.

**Socio-systemic branch (n=24)**

*Economic efficiency (n=10)*

Cabanilla, L.S., 2007. Socio-economic and political concerns for GM foods and biotechnology adoption in the Philippines. AgBioForum 10, 178–183.

Chong, M., 2003. Acceptance of golden rice in the Philippine “rice bowl”. Nat. Biotechnol. 21, 971–972. doi:10.1038/nbt0903-971

Dawe, D., Robertson, R., Unnevehr, L., 2002. Golden rice: What role could it play in alleviation of vitamin A deficiency? Food Policy 27, 541–560. doi:10.1016/S0306-9192(02)00065-9

Dawe, D., Unnevehr, L., 2007. Crop case study: GMO golden rice in Asia with enhanced vitamin A benefits for consumers. AgBioForum 10, 154–160.

De Steur, H., Mogendi, J.B., Blancquaert, D., Lambert, W., Van Der Straeten, D., Gellynck, X., 2014. Genetically Modified Rice with Health Benefits as a Means to Reduce Micronutrient Malnutrition. Global Status, Consumer Preferences, and Potential Health Impacts of Rice Biofortification., in: Wheat and Rice in Disease Prevention and Health. pp. 283–299. doi:10.1016/B978-0-12-401716-0.00021-0

Hessler, K., 2011. Agricultural Biotechnology and Environmental Justice: Golden Rice as a Case Study. Environ. Ethics.

Kowalski, S.P., 2015. Golden Rice, Open Innovation, and Sustainable Global Food Security. Ind. Biotechnol. 11, 84–90. doi:10.1089/ind.2015.1506

Qaim, M., 2011. Genetically Modified Crops and Global Food Security. Front. Econ. Glob. 10, 29–54. doi:10.1108/S1574-8715(2011)0000010007

Stein, A.J., Sachdev, H.P.S., Qaim, M., 2008. Genetic Engineering for the Poor: Golden Rice and Public Health in India. World Dev. 36, 144–158. doi:10.1016/j.worlddev.2007.02.013

Zimmermann, R., Qaim, M., 2004. Potential health benefits of Golden Rice: A Philippine case study. Food Policy 29, 147–168. doi:10.1016/j.foodpol.2004.03.001

*Equity and holism (n=14)*

Brooks, S., 2011. Is international agricultural research a global public good? The case of rice biofortification. J. Peasant Stud. 38, 67–80. doi:10.1080/03066150.2010.538581

Egana, N.E., 2003. Vitamin A Deficiency and Golden Rice-A Literature Review. J. Nutr. Environ. Med. 13, 169–184. doi:http://dx.doi.org/10.1080/13590840310001619414

Fuchs, D., Glaab, K., 2011. Material power and normative conflict in global and local agrifood governance: The lessons of “Golden Rice” in India. Food Policy 36, 729–735. doi:10.1016/j.foodpol.2011.07.013

Johns, T., Eyzaguirre, P.B., 2007. Biofortification, biodiversity and diet: A search for complementary applications against poverty and malnutrition. Food Policy 32, 1–24. doi:10.1016/j.foodpol.2006.03.014

Krawinkel, M.B., 2009. b-Carotene from rice for human nutrition? Am. J. Clin. Nutr. 695–696. doi:10.3945/ajcn.2009.28175.b

Krawinkel, M.B., 2007. What we know and don’t know about Golden Rice. Nat. Biotechnol. 25, 623; author reply 624. doi:10.1038/nbt0607-623

Liu, Y. liang, 2006. Exploration of the Biopolitics of GMOs: Using Golden Rice as an Analytical Model. Agric. Sci. China 5, 885–894. doi:10.1016/S1671-2927(07)60001-7

Lorch, A., 2001. Is this the way to solve malnutrition? Biotechnol. Dev. Monit.

Monastra, G., Rossi, L., 2003. Transgenic Foods as a Tool for Malnutrition Elimination and Their Impact on Agricultural Systems. Riv. di Biol. - Biol. Forum 96, 363–384.

Nestle, M., 2001. Genetically engineered “golden” rice unlikely to overcome vitamin A deficiency. J. Am. Diet. Assoc. doi:10.1016/S0002-8223(01)00073-6

Scott, D., 2011. The Technological Fix Criticisms and the Agricultural Biotechnology Debate. J. Agric. Environ. Ethics 24, 207–226. doi:10.1007/s10806-010-9253-7

Small, E., 2014. 46. Golden Rice – a food fight to enhance the unsustainable monarch of mega-crops. Biodiversity 15, 269–289. doi:10.1080/14888386.2014.963145

Stone, G.D., Flachs, A., 2014. The problem with the farmer’s voice. Agric. Human Values 31, 649–653. doi:10.1007/s10460-014-9535-1

Stone, G.D., Glover, D., 2016. Disembedding grain: Golden Rice, the Green Revolution, and heirloom seeds in the Philippines. Agric. Human Values. doi:10.1007/s10460-016-9696-1
